# Gclust: A Parallel Clustering Tool for Microbial Genomic Data

**DOI:** 10.1016/j.gpb.2018.10.008

**Published:** 2020-01-07

**Authors:** Ruilin Li, Xiaoyu He, Chuangchuang Dai, Haidong Zhu, Xianyu Lang, Wei Chen, Xiaodong Li, Dan Zhao, Yu Zhang, Xinyin Han, Tie Niu, Yi Zhao, Rongqiang Cao, Rong He, Zhonghua Lu, Xuebin Chi, Weizhong Li, Beifang Niu

**Affiliations:** *1* Computer Network Information Center, Chinese Academy of Sciences, Beijing 100190, China; *2* University of Chinese Academy of Sciences, Beijing 100190, China; *3* Center of Scientific Computing Applications & Research, Chinese Academy of Sciences, Beijing 100190, China; *4* Guizhou University School of Medicine, Guiyang 550025, China; *5* J. Craig Venter Institute, La Jolla, CA 92037, USA

**Keywords:** Microbial genome clustering, Parallelization, Sparse suffix array, Maximal exact match, Segment extension

## Abstract

The accelerating growth of the public microbial genomic data imposes substantial burden on the research community that uses such resources. Building databases for non-redundant reference sequences from massive microbial genomic data based on clustering analysis is essential. However, existing clustering algorithms perform poorly on long genomic sequences. In this article, we present Gclust, a parallel program for clustering complete or draft genomic sequences, where clustering is accelerated with a novel **parallelization** strategy and a fast sequence comparison algorithm using **sparse suffix arrays** (SSAs). Moreover, genome identity measures between two sequences are calculated based on their **maximal exact matches** (MEMs). In this paper, we demonstrate the high speed and clustering quality of Gclust by examining four genome sequence datasets. Gclust is freely available for non-commercial use at https://github.com/niu-lab/gclust. We also introduce a web server for clustering user-uploaded genomes at http://niulab.scgrid.cn/gclust.

## Introduction

The first complete bacterial genome was published more than 20 years ago [1]. During the last decade, the number of sequenced genomes has been growing very rapidly, mainly due to the development of low cost and high throughput DNA sequencing technologies [2]. As of the beginning of 2018, the Genomes OnLine Database (GOLD; https://gold.jgi.doe.gov/) has collected data from more than 180 thousand sequencing projects. Most genomic studies have been focusing on microbial species, especially bacteria. Thus, the growth of publically available bacterial genomes have become substantial and the amount of such data pose significant challenges for researchers interested in using these resources efficiently. In addition, these databases host a large portion of redundant genomes from the same or closely related species and the redundancy has to be reduced.

Clustering algorithms are key for redundancy reduction and there have been many of them available including CD-HIT [3], UCLUST [4], DNACLUST [5], canopies [6], Linclust [7], CLOSET [8], and SynerClust [9], among others. Most of them are efficient at clustering DNA sequences from hundreds to a few thousands of base pairs, including expressed sequence tags (ESTs), short reads from the next generation sequencers, and amplicon sequences, but less efficient on longer sequences. In fact, these programs are not able to handle typical bacterial genomes of mega basepairs in size. The performances and features of these clustering programs have been reviewed in many publications, such as this recent report [10].

BLASTclust from the BLAST package [11] can be used for clustering long sequences, but it is too slow to process large-scale genomic sequences. Other genome alignment tools, such as MUMmer [12], BLASTZ [13], and Mauve [14], are also incapable of clustering large-scale genomic sequences, because they were originally designed to assess genomic variation and rearrangements by pairwise or multiple alignment of a small number of genomes.

Since sequence clustering is time-consuming, most clustering programs use different algorithms to improve performance. For example, CD-HIT [3] uses a heuristic based on short word filtering to reduce computational load. Beside short word index tables or hash tables, suffix trees and suffix arrays have also been widely used for sequence comparison. For example, Malde et al. [15] introduced an EST clustering algorithm, where sub-quadratic time complexity was achieved by using suffix arrays. Another strategy to reduce the overall computational time in clustering a large dataset is through parallelization. For example, a multi-threaded function was introduced in an enhanced version of CD-HIT [16], and this was able to achieve quasi-linear speedup when using up to 8 cores.

In this article, we introduce Gclust, a fast program for clustering microbial genomic sequences. A key algorithm in Gclust for sequence comparison is based on sparse suffix arrays (SSAs). Our method has several key features. First, it is specially designed for clustering very long sequences, of up to typical prokaryotic genomes. Genomic sequences are compared using extended maximal exact matches (MEMs), which are used to calculate genome sequence identity. Second, a fast algorithm was implemented in building SSAs and querying SSAs to identify MEMs. Third, Gclust supports multi-threaded parallel computing.

## Method

### Datasets

We used four genomic sequence datasets (Table 1) from NCBI to test the performance of Gclust. The first three datasets contain viral, archaeal, and fungal genomic data (ftp://ftp.ncbi.nlm.nih.gov/refseq/release/). The bacterial genomes in the fourth dataset were selected from the NCBI RefSeq genome list (ftp://ftp.ncbi.nlm.nih.gov/genomes/refseq/assembly_summary_refseq.txt) according to the following criteria: (1) genomes assembled at only contig level were excluded; (2) all the NCBI reference genomes and representative genomes were included; and (3) the remaining genomes were included if assembled to complete genomes or chromosomes.

**Table 1:** **Detailed information for the four genomic datasets analyzed using Gclust**

**Dataset**	**Size (Mbp)**	**No. of sequences**	**Sequence length (bp)**		
			**Maximal**	**Minimal**	**Average**
Viral	261	9578	2,473,870	200	27,290
Archaea	2028	38,381	5,751,492	22	52,845
Fungi	7213	79,365	11,880,248	86	90,879
Bacteria	19,848	112,111	13,033,779	69	17,046

*Note*: Sequences shorter than 21 bp were discarded.

### Implementation

Gclust is implemented in C and C++ and POSIX threads programming (https://computing.llnl.gov/tutorials/pthreads/) is used for parallelization. We also used the SeqAn [17] and libdivsufsort libraries (https://github.com/y-256/libdivsufsort) in the implementation.

### Preliminaries

A key problem in sequence comparison is pattern matching between sequences. Similar sequences can be detected by common fragments. MEMs are exact matches between two strings that cannot be extended without a gap [18]. The classical approach to find MEMs between a pair of sequences is to use suffix trees and search for maximal matching blocks [19]. However, suffix trees require about ten to twenty times the memory of the source text, even in optimal implementations.

In order to reduce the memory cost in finding MEMs, Manber and Myers [20] adopted suffix arrays (SAs), a data structure that is a sorted list of all the suffixes of a large text. Later, enhanced suffix arrays (ESAs) replaced suffix trees, since the use of suffix trees often bottlenecked large scale applications [21]. Khan et al. [18] introduced another method, where SSAs were used to find MEMs. Recently, another SSA-based tool, essaMEM, has been reported [22]. Compared to full-text suffix arrays, sparse suffix arrays store every *K*-th suffix of the text and occupy much less memory.

The variable declaration is as follows: d:[s...e] and q:[l...r] are the intervals of query sequence *P*. SA(i) is the *i-*th value of the suffix array. sn(p) is the serial number of P. Location(SA(i)) is the serial number of the sequence which includes the *i-*th suffix SA(i). *LCP* means the longest common prefix, and *LCP[i]* is the *i*-th value of the *LCP* array.

In order to find MEMs using SSAs, we adapted the method suggested by Khan et al. [18]. MEMs are found according to two intervals (d:[s...e] and q:[l...r], where q:[l...r] is a subinterval of d:[s...e]) and is obtained by a top-down binary search. There are two cases whereby MEMs between *S* and *P* can be found (where *S* is the reference sequence and *P* is the query sequence). The first case is when at most $L-\left(K-1\right)$ characters in length are found and the match can be recovered by scanning the region between sparsely indexed suffix positions. Here *K* is the sparse step of the suffix array and *L* is the only constraint of the minimum length of MEMs. The other case is when at least $d⩾L-\left(K-1\right)$ matched characters are found, and the two intervals are used to determine the length and position of MEMs.

### Gclust algorithm

Gclust is a greedy incremental clustering algorithm for genomic sequences. The algorithm is explained with pseudo-codes (Table 2).

**Table 2:** **Pseudo-codes of the Gclust algorithm**

The main Gclust parallel algorithm includes (1) sorting the input genome sequences from long to short and (2) dividing the input genome sequences into blocks based on the memory occupied by suffix arrays and process these blocks one after another.

For each block, the following steps are performed. (a) one suffix array is constructed before clustering using the representative sequences. The longest sequence is automatically classified as the first representative sequence within the block. (b) Each remaining query sequence is searched in the suffix array and is compared to the existing representative sequences longer than it. The comparison is made by attempting to extend MEMs. If the MEM-based sequence identity satisfies the user-specified clustering threshold, the query sequence is considered redundant, or is otherwise a new representative sequence. (c) A new suffix array is reconstructed using all the representative sequences found in this block. This new suffix array is used in comparing sequences in the remaining blocks to the representative sequences in this block in parallel to identify redundant sequences. (d) The main loop of the algorithm processes the next block with steps (a) through (c) until all blocks are processed.

### Segment match refinement and extension

**Figure 1:**
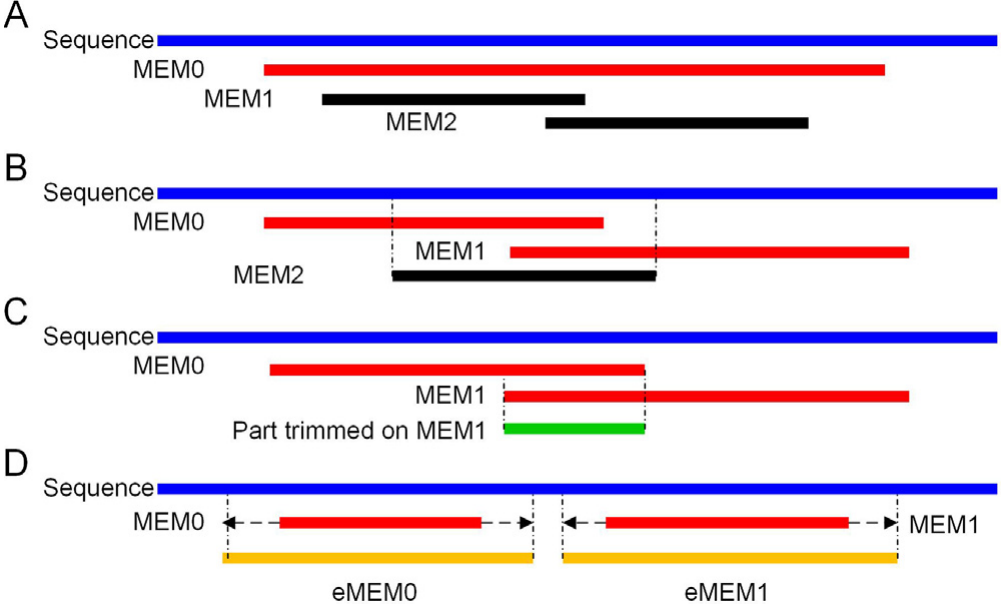
**MEM refinement and extension process** The plot represents four sub-procedures in the process: removing MEMs (in black) whose coordinates on a representative sequence (or query sequence; in blue) are completely included in a larger MEM (in red) (**A**) or in two neighboring MEMs (in red) (**B**), trimming the remaining MEMs (in red, *e.g.*, MEM1 in sub-procedure C) of a representative sequence (or query sequence; in blue) that exhibit partial overlap (in green) (**C**), and extending the MEMs retained after refinement using the given score matrix (**D**) to obtain the respective eMEMs. MEM, maximal exact match; eMEM, extended MEM.

Given sequences *A* and *B* and a set *Σ* of matched segments between them, the matched sequence problem is to compute a set of non-intersecting matches *Σ'* that are all sub matches of *Σ*, that maximize the amount of sequence covered by the matched segments. Halpern et al. [23] introduced an efficient method for refining a set of matched segments in which the projections of resulting segments onto each sequence were disjoint or identical. However, the method is time-consuming. Since a MEM spans the same length on the two sequences being compared, it is less complicated to refine the MEMs. Deloger et al. [24] designed an approximate solution for computing the maximal unique matches index (MUMi). Here, we used a similar solution to refine MEMs and to compute the sequence identity using refined MEMs (Figure 1).

The procedures are as follows. (1) MEMs whose coordinates on are presentative sequence (or query sequence) are completely included in a larger MEM are removed, *e.g.*, MEM1 and MEM2 in Figure 1A. (2) MEMs whose coordinates on a representative sequence (or query sequence) are completely included in two neighboring MEMs are removed, *e.g.*, MEM2 in Figure 1B. (3) The remaining MEMs of a representative sequence (or query sequence) that exhibit partial overlap are trimmed. To do this, MEMs are sorted according to their beginning positions on a representative sequence (or query sequence). Starting from the last element of the list, each MEM is compared to its leftward neighbor. In cases of overlap, the left end of the current MEM is trimmed, *e.g.*, MEM1 in Figure 1C, *i.e.*, its end coordinates on both the representative and query sequences are shifted rightward so that no overlap exists on the representative sequence (or query sequence) (Figure 1C). (4) The MEMs retained are extended after refinement using the given score matrix (Figure 1D). While computing the MEM extension, the score matrix is used to give a reward or penalty. We determine the identity between two sequences based on the extended MEMs (eMEMs). This eMEM identity (eMEMi) is calculated using the following formula:

(1) $$\mathrm{eMEMi}=\;N_{\mathrm{match}}\text{/}L_{\mathrm{query}}$$

$N_{\mathrm{match}}$ is the number of matched nucleotides within extended MEMs and $L_{\mathrm{query}}$ is the length of shorter sequences. The lengths of the representative sequences are always longer than that of the query sequences, because the sequences are sorted by length in descending order. Thus, eMEMi is used to measure the identity between the representative and the query sequence. Formula (1) relies on the choice of minlen, which is the minimal size of the exact matches to be included in MEMs. We extend the MEMs by using a function from the SeqAn [17] library. In SeqAn, alignments allow the insertion of gaps into sequences through extension. SeqAn uses a seed-and-extend algorithm to realize extension. In the un-gapped cases, matches and mismatches are assigned with scores; these scores are then summed up and the running total will drop when one or more mismatches occur. In the gapped cases, gaps will be created with negative scores (http://seqan.readthedocs.io/en/master/Tutoral/Algorithms/SeedExtension.html#tutorial-algorithms-seed-extension). While computing the MEM extension, the score matrix is used to give a reward or penalty. The minlen value is determined empirically. It has been reported that under the uniform Bernoulli model, no maximum unique matches (MUMs) longer than 21 are expected by chance in 1.7-Mbp random genomes [25]. This suggests that a minlen value of 21 can avoid many spurious matches.

### Using suffix arrays to find MEMs

For a given sparse step *K*, there are two major drawbacks in finding MEMs using sparse suffix arrays: (1) the need to run the search procedure *K* times; and (2) a complicated search procedure is required when the MEM is shorter than *K*. In Gclust, we only use *K* = 1 for small MEMs to decrease the cost of repeated searches. For longer MEMs, we use *K* = 2–4. Since MEMs shorter than 21 are unlikely to find redundant sequences, our choice of *K* avoids the second drawback. For a large MEM or a clustering of 100% identity, a larger *K* will shorten the time in constructing the suffix array with little impact on the efficiency of MEM searching.

However, unlike other mapping programs in which the suffix arrays for reference genomes are constructed only once prior to mapping, in Gclust, the suffix arrays for each block are constructed in real time. Therefore, it is important to accelerate the sorting process of suffix arrays within the block, especially when clustering at 100% identity, when the construction of suffix arrays becomes the most time-consuming step.

### Clustering within one genome block

According to the greedy incremental clustering algorithm, a sequence *S* only need to be searched against the sequences longer than *S* in the pre-constructed the suffix array for that block. Here we implemented a modified MEM filtering algorithm (*collectMEMs*). This approach avoids the need to scan up to *P* characters to the left of the match and then discarding the MEMs located in those sequences that are shorter than *S* (Table S1).

The algorithm to find MEMs using sparse suffix arrays by Khan et al. [18] relies on a traverse algorithm to match up to $L-\left(K-1\right)$ characters and to find the longest match. If a match of length $⩾L-\left(K-1\right)$ characters can be obtained, the suffix array interval d:[s...e] corresponding to matches of length $⩾L-\left(K-1\right)$ and the interval q:[l...r] corresponding to the longest match are used by the *collectMEMs* algorithm to find right maximal matches. Each right maximal match must be verified for the left by scanning up to K characters using the *findL* algorithm to the left of the match. In Gclust, for the sorted sequences S:[1…N] in one block, given the query sequence P, we modified the *collectMEMs* algorithm to discard the MEMs located on the sequences shorter than *S*.

### Parallelization techniques used

Three different explicit parallel extensions to the C language are the Message-Passing Interface (MPI), POSIX threads (Pthreads), and OpenMP [26]. MPI is used for distributed-memory programming. While OpenMP and Pthreads are both APIs for shared-memory programming, Pthreads is more flexible than OpenMP. Due to the advantages of using shared memory, in Gclust we adapted Pthreads to facilitate parallel processing of clustering.

The major part of the Gclust algorithm (Table 2) includes two primary alignment processes (intra- and inter-block). The main computation involves finding MEMs. Multiple query sequences need to be searched in the same suffix array. In Gclust, these are distributed to individual processors or cores.

## Results and discussion

The greedy incremental clustering algorithm introduced by the enhanced version of CD-HIT [16] was implemented in Gclust for clustering genomic sequences. In Gclust, genome identity measures of two sequences are calculated based on the extension of their MEMs. We implemented an improved SSA algorithm to find these MEMs.

We tested the performance of Gclust using four RefSeq genome datasets (viral, archaeal, fungal and bacterial genome data; Table 1). Tests were done on an Era supercomputer with a 24-core Intel(R) Xeon(R) CPU E5-2680 v3 @ 2.50 GHz with 256-GB RAM.

### Clustering performance

The cluster results for the four datasets are shown in Table 3. Genomes were clustered at 90% eMEMi. For the viral dataset, 9578 sequences were clustered into 9101 clusters. 38,381 archaea sequences were reduced to 16,064 non-redundant sequences, a reduction of 58%. The fungal and bacterial datasets were reduced by 13% and 6% respectively. It took Gclust less than two hours to cluster the 2-GB archaeal dataset. For the largest bacterial dataset, Gclust took 138.11 h on a single computer with 16 threads.

**Table 3:** **Clustering results and performance of Gclust at 90% eMEMi using 16 threads**

**Dataset**	**No. of sequences**	**No. of clusters**	**Running time (min)**
Viral data	9578	9101	8.7
Archaeal data	38,381	16,064	88.0
Fungal data	79,365	68,698	1322.8
Bacterial data	112,111	105,867	7678.8

*Note*: The parameters used for clustering are as follows: -minlen 41 -both -nuc -threads 16 -chunk 400 -loadall -memiden 90 -rebuild -ext 1 -sparse 4. Parameter “-both” indicates that Gclust compares both strands of DNA sequences. MEM, maximal exact match. eMEMi, extended MEM identity.

A comparison between Gclust and BLASTclust is shown in Table 4. Four smaller datasets, which contained subsets of the viral, archaeal, fungal and bacterial genomic data, were used to test the efficiency of Gclust relative to BLASTclust. Smaller datasets were used for this comparison to accommodate the long running time of BLASTclust. Our tests showed that Gclust was more than 35 times faster in the viral subset, more than 40 times faster in the archaeal subset and more than 300 times faster in the bacterial subset, and generated fewer clusters in all subsets except for the fungal subset. BLASTclust could not process the fungal subset since the longest sequence was beyond the limit of BLASTclust (Table 4).

**Table 4:** **Comparison of BLASTclust and Gclust**

**Dataset**	**Size (Mbp)**	**No. of sequences**	**Length of the longest sequence (Mbp)**	**Running time (s)**		**No. of clusters**	
				**BLASTclust**	**Gclust**	**BLASTclust**	**Gclust**
Viral subset	213	8584	2.474	10,075	245	8454	8215
Archaeal subset	192	4135	3.122	8148	224	4085	2364
Fungal subset	129	502	6.910	/	71	/	402
Bacterial subset	331	14,891	0.997	73,672	237	9206	2284

*Note*: The parameters used in Gclust are as follows: -minlen 21 -both -nuc -threads 8 -rebuild -loadall -memiden 90; the parameters used in BLASTclust are as follows: -a 8 -p F -L 0.1 -b F -S 90. “/” means that BLASTclust could not process the fungal subset because the longest sequence was too long.

**Figure 2:**
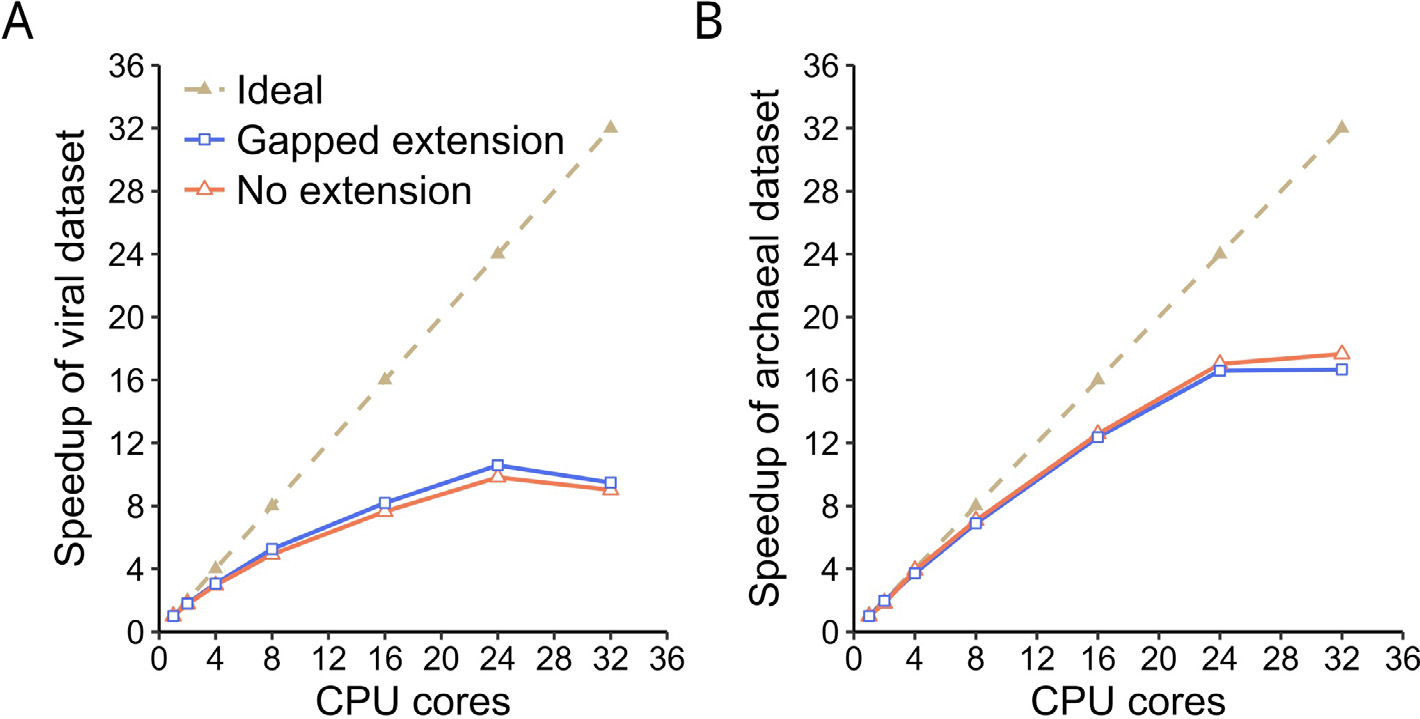
**The speedup of parallel Gclust for the viral and archaeal genome datasets** The plot represents the average of 4 runs for speedup clustering of viral (**A**) and archaeal datasets (**B**), respectively.

Gclust applies a parallel strategy that is similar to that introduced by the multi-threaded version of CD-HIT [16]. Using the viral and archaeal genomic datasets, we tested the parallelization of Gclust when using multiple compute cores (Figure 2). The greedy incremental clustering procedure used by Gclust (see Method) is intrinsically sequential, so it is not feasible to reach linear speedup with parallelization. Here, Gclust is able to achieve an eightfold speedup with 16 cores.

**Figure 3:**
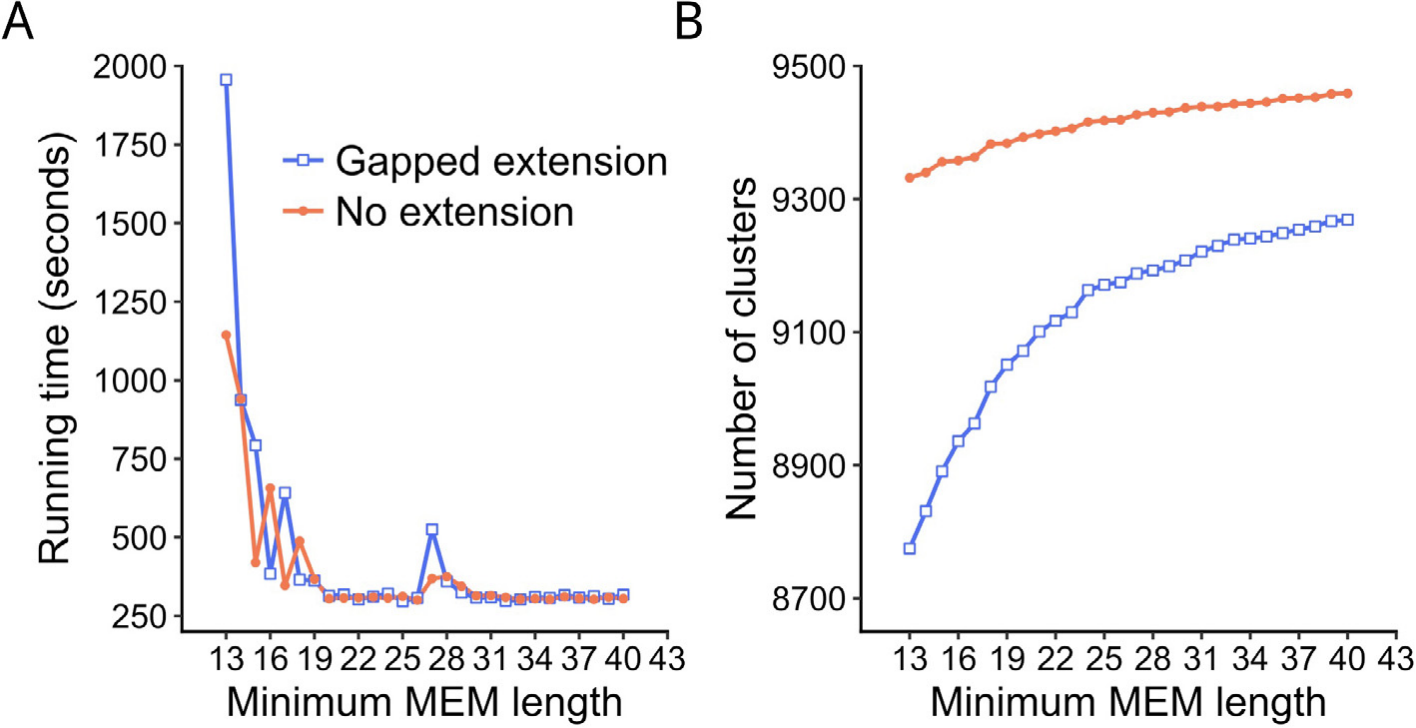
**Comparison of running time and the number of clusters with different minimal MEMs** Running time (**A**) and number (**B**) of clusters with the minimal MEM length varying from 13 to 40 of the viral dataset.

Minimal MEM length is a key parameter in Gclust and affects both running time and the number of clusters found. The default minimal MEM length in Gclust is 21. The selection of this default value is described in the Method. Here, using the viral genomic dataset, we tested different MEM lengths from 13 to 40 in gapped and non-gapped extension cases (Figure 3).

In non-gapped extension cases, a sequence is rejected if its alignment score is very low, and this is much faster than gapped extension (Figure 3A). Given the same minimum MEM length, the number of clusters in gapped extension cases is always smaller than in non-gapped extension cases since the algorithm identifies more redundant sequences with gapped extension (Figure 3B).

### Efficiency of MEM identification

In Gclust, finding MEMs is the most time-consuming step. We therefore adapted a fast, lightweight suffix array sorting algorithm and modified the search algorithm to find MEMs. To evaluate its effectiveness, we compared Gclust and MUMmer3 in finding MEMs. In all test cases, Gclust was considerably faster than MUMmer3 (Table S2).

When suffix array requires too much memory, sparse suffix arrays (that use a sparse step *K*) are usually used to reduce memory demand by sacrificing the accuracy of clustering. With a higher *K*, some redundant sequences might be missed.

However, for larger MEMs, especially given a higher clustering threshold (*e.g.*, 100% eMEMi), sparse steps significantly reduce the total clustering time without sacrificing accuracy. We tested the performance of Gclust with different sparse steps at 100% eMEMi using viral and fungal genomes (Table S3) and observed shorter runtime with larger *K* (≤4). The number of clusters was consistent across all sparse steps.

## Conclusion

In this paper, we present an open source program for clustering microbial genomic sequences. This algorithm provides many options for users to control the clustering process, for example, the optimal sparse step parameter *K*. We show that our method is efficient for large-scale genomic sequences with high accuracy. We expect that our parallelization strategy can be further optimized and improved to achieve better scalability.

## Availability

Gclust is freely available for non-commercial use at https://github.com/niu-lab/gclust. A web server for clustering user-uploaded genomes is available at http://niulab.scgrid.cn/gclust. The four datasets for viral, archaea, fungi, and bacteria were deposited in RefSeq of NCBI and can be accessed at ftp://ftp.ncbi.nlm.nih.gov/refseq/release/.

## Authors’ contributions

BN and WL conceived the idea and designed the study. RL performed data analysis. BN and WL analyzed the results and drafted the manuscript. CD built the webserver. RL and HZ contributed the code debugging. XH, XYL, WC, XL, DZ, YZ, ZL, and XC edited and revised the manuscript. TN, YZ, RC, and RH provided technical support for the test environment. XH designed and drew the figures. All authors read and approved the final manuscript.

## Competing interests

The authors declare that no competing interests exist.

## Supplementary data

Supplementary Table S1 Pseudo-codes of the modified *collectMEMs* algorithm.

Supplementary Table S2 Comparison between Gclust and MUMmer3 in finding MEMs.

Supplementary Table S3 Total running time of clustering at 100% eMEMi.
