# Supplementary material for: Gclust: A Parallel Clustering Tool for Microbial Genomic Data
Source: Genomics Proteomics Bioinformatics. 2020 Jan 7;17(5):496–502. doi: 10.1016/j.gpb.2018.10.008 (PMC7056916; doi:10.1016/j.gpb.2018.10.008)
Supplement: Supplementary Table S1 — Pseudo-codes of the modified collectMEMs algorithm. [file mmc1.docx]

**Table S1 Pseudo-codes of the modified *collectMEMs* algorithm**

| Algorithm 2: *collectMEMs* ($\text{P, d: }\left[ \text{s...e} \right]\text{, q: }\left[ \text{l...r} \right]\text{, sn (P)}$) |
| --- |
| 1: **for** $\text{i=l...r}$ **do**  2: **if** $\text{Location (SA(i))<sn (P)}$ **then** *findL*($\text{p, SA[i], q}$)  3: **while** $\text{q>=d}$  4: **if** $\text{r+1<n/K}$ **then** $\text{q=max(LCP[l], LCP[r+1]})$  5: **else** $\text{q=LCP[l]}$  6: **if** $\text{q>=d}$ **then**  7: **while** $\text{LCP(l)>=q}$ **do**  8: $\text{l=l-1}$,  9: **if** $\text{Location(SA(l))<sn(P)}$  10: **then** *findL*($\text{P, SA[l], q}$)  11: **while** $\text{r+1<n/K}$ and $\text{LCP(r+1)>=q}$ **do**  12: $\text{r=r+1}$,  13: **if** $\text{Location(SA(r))<sn(P)}$  14: **then** *findL*($\text{P, SA[r], q}$) |
