# Supplementary material for: Gclust: A Parallel Clustering Tool for Microbial Genomic Data
Source: Genomics Proteomics Bioinformatics. 2020 Jan 7;17(5):496–502. doi: 10.1016/j.gpb.2018.10.008 (PMC7056916; doi:10.1016/j.gpb.2018.10.008)
Supplement: Supplementary Table S2 — Comparison between Gclust and MUMmer3 in finding MEMs. [file mmc2.docx]

**Table S2 Comparison between Gclust and MUMmer3 in finding MEMs**

| **Dataset** | **Size of the reference sequence (Mbp)** | **Size of the query sequence (Mbp)** | **Running time (s)** | |
| --- | --- | --- | --- | --- |
|  |  |  | **MUMmer 3** | **Gclust** |
| Viral subset | 2.5 | 2.2 | 2 | 1.5 |
| Archaeal subset | 5.8 | 5.4 | 6 | 4.5 |
| Fungal subset | 11.9 | 11.7 | 15.25 | 12.5 |
| Bacterial subset | 14.8 | 13.0 | 18 | 16.75 |

*Note*: The parameters used in Gclust are as follows: -minlen 21 -both -nuc -threads 8 -loadall -memiden 90 -rebuild -sparse 2; the parameters used in MUMmer3 are as follows: -n -l 21 -b -s -c -L. Results are average of four test runs. The reference and query sequences are the longest and the second longest genomes in the datasets, respectively.
