# Supplementary material for: Gclust: A Parallel Clustering Tool for Microbial Genomic Data
Source: Genomics Proteomics Bioinformatics. 2020 Jan 7;17(5):496–502. doi: 10.1016/j.gpb.2018.10.008 (PMC7056916; doi:10.1016/j.gpb.2018.10.008)
Supplement: Supplementary Table S3 — Total running time of clustering at 100% eMEMi. [file mmc3.docx]

**Table S3 Total running time of clustering with 100% eMEMi**

| **Dataset** | **Size (Mbp)** | **No. of sequences** | ***K* = 1** | |  | ***K* = 2** | |  | ***K* = 3** | |  | ***K* = 4** | |
| --- | --- | --- | --- | --- | --- | --- | --- | --- | --- | --- | --- | --- | --- |
|  |  |  | **Running time (s)** | **No. of clusters** |  | **Running time (s)** | **No. of clusters** |  | **Running time (s)** | **No. of clusters** |  | **Running time (s)** | **No. of clusters** |
| Viral data | 261 | 9578 | 198 | 9563 |  | 104 | 9563 |  | 76 | 9563 |  | 67 | 9563 |
| Archaeal data | 2028 | 38,381 | 1384 | 31,350 |  | 706 | 31,350 |  | 768 | 31,350 |  | 500 | 31,350 |
| Fungal data | 7213 | 79,365 | 3394 | 78,351 |  | 2242 | 78,351 |  | 1801 | 78,351 |  | 1713 | 78,351 |

*Note*: *K* designates sparse step of suffix array. The parameters used in Gclust are as follows: -minlen 21 -both -nuc -threads 8 -loadall -memiden 100 -rebuild -ext 1.
